# Pharmaceutical Products and Pesticides Toxicity Associated with Microplastics (Polyvinyl Chloride) in *Artemia salina*

**DOI:** 10.3390/ijerph182010773

**Published:** 2021-10-14

**Authors:** María Gemma Albendín, Vanessa Aranda, María Dolores Coello, Carmen González-Gómez, Rocío Rodríguez-Barroso, José María Quiroga, Juana María Arellano

**Affiliations:** 1 Toxicology Laboratory, University Institute of Marine Research (INMAR), International Campus of Excellence of the Sea (CEI MAR), Faculty of Marine and Environmental Sciences, University of Cádiz, 11510 Cádiz, Spain; gemma.albendin@uca.es (M.G.A.); vanessa.arandaquiros@alum.uca.es (V.A.); mgong45@oc.mde.es (C.G.-G.); juana.arellano@uca.es (J.M.A.); 2 Environmental Technologies Department, University Institute of Marine Research (INMAR), International Campus of Excellence of the Sea (CEI MAR), Faculty of Marine and Environmental Sciences, University of Cádiz, 11510 Cádiz, Spain; rocio.rodriguez@uca.es (R.R.-B.); josemaria.quiroga@uca.es (J.M.Q.)

**Keywords:** microplastics, simvastatin, carbamazepine, chlorpyrifos, triclosan, cholinesterase activity

## Abstract

Pharmaceutical products, as well as insecticides and antimicrobials, have been extensively studied, but knowledge of their effects—especially those caused by their mixtures with microplastics—on aquatic organisms remains limited. However, it should be borne in mind that the state of knowledge on acute and chronic effects in aquatic organisms for pharmaceuticals and pesticides is not similar. In response, this investigation analyzed the presence of microplastics (polyvinyl chloride) and their impacts on the toxicity of chlorpyrifos (an insecticide) and triclosan (an antibacterial) when they coincide in the environment, alongside the two most consumed drugs of their type (hypolipemic and anticonvulsant, respectively), namely simvastatin and carbamazepine, in *Artemia salina*. LC_50_ and cholinesterase enzyme activity were calculated to determine the possible neurotoxicity associated with emergent contaminants in the treatments. The LC_50_ values obtained were 0.006 mg/dm^3^ for chlorpyrifos, 0.012 mg/dm^3^ for chlorpyrifos associated with microplastics, 4.979 mg/dm^3^ for triclosan, 4.957 mg/dm^3^ for triclosan associated with microplastics, 9.35 mg/dm^3^ for simvastatin, 10.29 mg/dm^3^ for simvastatin associated with microplastics, 43.25 mg/dm^3^ for carbamazepine and 46.50 mg/dm^3^ for carbamazepine associated with microplastics in acute exposure. These results indicate that the presence of microplastics in the medium reduces toxicity, considering the LC_50_ values. However, exposure to chlorpyrifos and carbamazepine, both alone and associated with microplastics, showed a decline in cholinesterase activity, confirming their neurotoxic effect. Nevertheless, no significant differences were observed with the biomarker cholinesterase between the toxicant and the toxicant with microplastics.

## 1. Introduction

Currently, aquatic systems, in some cases, show a significant deterioration. Human intervention is one of the sources of water pollution; domestic, industrial and agricultural activities use and produce large quantities of substances such as insecticides, antibacterials, plastics, drugs, etc. This complex mixture of substances with plastics could be responsible for environmental pollution, since aquatic/sediment/soil organisms could be especially exposed [1,2,3]. Plastic waste in the environment is an increasing problem that has generated a lot attention from both researchers and the general public. This debris fragments in the environment into smaller particles, such as microplastics (MP; <5 mm), whose presence has been detected until in the Atlantic gyre [4].

The presence of pharmaceuticals and their metabolites in groundwater, lakes, rivers, oceans and so forth has been confirmed by authors such as Liu et al. (2011) [5], Biel-Maeso et al. (2018) [6], Barbosa et al. (2019) [7] and Primrose et al. (2019) [8]. High concentrations of simvastatin and carbamazepine have been observed in the aquatic environment due to the progressive increase in their consumption in recent decades. Simvastatin is the most consumed hypolipemic drug in Spain, having increased by 670% since 2000 [9]. Similarly, carbamazepine is the most widely used anticonvulsant, with an increase in consumption of 41.52% since 2008 [10].

The high consumption of simvastatin and carbamazepine and their subsequent discharge into urban wastewater through human excretion, drug residues that are not properly disposed of and waste generated during their manufacture cause large quantities of drugs and their metabolites to reach wastewater stations (WWTPs) [11]. These toxic substances are not completely eliminated during treatment processes. Due to their high cost, most WWTPs do not have purification systems capable of totally eliminating pharmaceutical compounds. Instead, the effluent is typically dumped, and high concentrations of these products are maintained in aquatic ecosystems, where they can have toxic impacts on organisms [11]. Neuparth et al. (2014) [12] have shown that exposure to simvastatin disrupts the reproduction and growth of the amphipod crustacean *Gammarus locusta* in vivo. In vitro research has also indicated that this compound and its metabolite cause liver cytotoxicity in rainbow trout (*Oncorhynchus mykiss*), metabolic inhibition and loss of cell membrane integrity [13]. Carbamazepine has been found to produce harmful effects in organisms, such as moult and growth inhibition in the crustacean *Eriocheir sinensis* [14]; malformations during embryonic development in *Mytilus galloprovincialis* [15]; and neurotoxic effects, such as alterations in mobility and behavior, in *Danio rerio* embryos [16].

Today, a wide range of pesticides are used in agriculture and horticulture and play a key role in global food production. The main factors driving the increase in pesticide use are related to population growth, necessitating the intensified exploitation of agricultural land [17].

Chlorpyrifos (CPF) is an organophosphate that is used to control foliage and insect and arthropod pests in the soil in a variety of foods [18]. In 2019, the European Food Safety Authority confirmed a human health concern due to the possible genotoxicity and neurotoxicity of CPF. Previously, CPF had been banned in Hawaii and California [19], and it is no longer authorized for use in plant protection products in the EU from 2020. CPF is a typical insecticide that is difficult to dissolve in water (1.4 mg/dm^3^ at 25 °C). It can be transported from groundwater by leaching and travel in surface water via runoff and erosion [20,21]. Low concentrations of CPF in the aquatic environment cause adverse effects, such as histopathological and behavioral changes, oxidative stress, neurological damage and genotoxicity in algae, fish, crustaceans and shellfish [19].

In the group of antibacterials, triclosan (TCS) is found in the composition of many products used in our daily lives, such as soaps, shampoos, detergents, deodorants, household items, additives in packaging and textiles. TCS has antibacterial and fungicidal properties and is released into the sewage network, eventually reaching sewage treatment plants that empty into rivers and seas. TCS represents a major source of pollution [22] because exposure leads to toxicity in aquatic organisms [23]. The routes of entry of TCS in humans are through skin contact, mucous membranes and the gastrointestinal tract, because this compound is rapidly metabolized and excreted through urine. It has a low probability of generating toxic effects, whether genotoxic, teratogenic, mutagenic or carcinogenic. However, TCS has structural similarities to thyroid hormones and androgenic and estrogenic disruptors [24]. Its mechanism of action as an antibacterial is based on disrupting the membrane through blocking lipid synthesis. It inhibits the transport protein enoyl-acylreductase, which participates in the biosynthesis of fatty acids necessary for the formation of cell membranes and in the reproduction of bacteria [25].

Microplastics (MPs) constitute another emerging contaminant that has been gaining relevance in recent years. Due to their low cost and versatility, plastics are increasingly in demand, having replaced conventional materials (paper, glass, wood and metal). In 2018, the production of plastics worldwide reached 360 million tons, including 62 million tons in Europe. The amount of plastics generated in the world has been growing exponentially for decades, from 348 to 359 million tons during the period 2017/2018 [26].

Some plastic products have a useful life of less than one year, while others have a durability of more than 50 years, but one thing that all of them have in common is that, when their useful life is over, they become waste. The disposal of many of these plastic wastes is not properly managed, so they end up contaminating aquatic ecosystems [27]. Although there are many types of marine debris (e.g., paper, glass and metal), plastics represent 82% of this waste [28]. This plastic garbage can be classified according to its size into macroplastics and microplastics, the latter representing the majority of the total garbage released into the marine environment. As is also the case with pharmaceutical substances, MPs originated by human activity in the form of textile fibers, remains of cosmetics and industrial processes end up in the sewage system and reach the marine environment through the effluents of WWTPs, where they are not completely eliminated [29,30,31].

They can also act as vectors for the transportation of other pollutants, because they have additives in their composition that can be released into the environment during the fragmentation process and they have the ability to adsorb other chemical pollutants on their surfaces, including heavy metals [32], hydrophobic organic pollutants and pharmaceutical compounds [33]. From this capacity of MPs to act as vectors for other contaminants derives the importance of investigating the interaction between MPs, pharmaceutical compounds and pesticides, as these contaminants are increasingly present in aquatic ecosystems. Authors such as Rainieri et al. (2018) [34] and Webb et al. (2020) [35] have shown that the presence of MPs in the environment affects the toxicity of other pollutants.

Cholinesterase (ChE) activity inhibition is one of the biomarkers of exposure, which is widely used and recognized as a biomarker to organophosphate compounds, such as CPF [36], as well as to pharmaceutical substances. In crustaceans, Thi et al. (2009) [37] showed that the cholinesterase activity present in adult *Penaeus monodon* was inhibited by the action of antibiotics. Albendín et al. (2021) [38] and Soto-Mancera et al. (2020) [39] determined a decreased level of cholinesterase activity exposed to organophosphate compounds in seabream. Nunes et al. (2016) [40] found that the anxiolytic diazepam inhibited the cholinesterase activity present in *Artemia parthenogenetica*. Similarly, Yu et al. (2018) [41] determined that MPs significantly reduced cholinesterase activity in *Eriocheir sinensis*.

Relative to fish and molluscs, the use of *Artemia salina* in toxicity tests has many advantages. These crustaceans have a shorter life cycle, provide available populations all year and offer both a standardized method of cultivation from dried cysts and easy handling. Therefore, *Artemia salina* has been successfully used in toxicological tests for decades [42,43,44].

For the above reasons, in this study, the impacts of the toxicity of MPs (polyvinyl chloride), pharmaceutical substances (simvastatin and carbamazepine) and pesticides (chlorpyrifos and triclosan) on *Artemia salina* were evaluated, in order to determine whether MPs in combination with drugs or pesticides increase the adverse effects on this organism. To measure possible adverse effects, toxicity tests were performed by determining the LC_50_, as well as the ChE, activity.

## 2. Material and Methods

### 2.1. Chemicals

Acetylthiocholine iodide (AcSCh) (Sigma-Aldrich, Merck KGaA, Darmstadt, Germany), 5,5′-dithiobis-(2-nitrobenzoic acid) (DTNB) (Merck, Merck KGaA, Darmstadt, Germany), acetone (Scharlab, Merck KGaA, Darmstadt, Germany), 5H-dibenzo[b,f]azepine-5-carboxamide (carbamazepine (CAS 298-46-4) (Sigma-Aldrich, Merck KGaA, Darmstadt, Germany),(1S,3R,7S,8S,8aR)-8-{2-[(2R,4R)-4-Hydroxy-6-oxotetrahydro-2H-pyran-2-yl]ethyl}-3,7-dimethyl-1,2,3,7,8,8a-hexahydro-1-naphthalenyl 2,2-dimethylbutanoate (simvastatin CAS 79902-63-9, Sigma-Aldrich), 5-Chloro-2-(2,4-dichlorophenoxy) phenol (triclosan (CAS 3380-34-5, Sigma-Aldrich) and O, O-dietil O-3,5,6-tricloro-2-piridil fosforotionato (chlorpyrifos) (CAS 2921-88-2, Sigma-Aldrich) were used. Bovine serum albumin (BSA) and the BioRad Protein Assay were supplied by BioRad (Madrid, Spain). Polyvinyl chloride (PVC) (CAS 9002-86-2, Aldrich. The data for the compound are listed in PubChem: https://pubchem.ncbi.nlm.nih.gov/compound/6338. (accessed on 13 September 2021), MP < 5 mm), potassium dichromate (K_2_Cr_2_O_7_) (CAS 7778-50-9, Panreac, AppliChem. ITW Reagents, Barcelona, Spain), di-sodium hydrogen phosphate anhydrous (CAS 7558-79-4, Merck, Darmstadt, Germany) and sodium dihydrogen phosphate monohydrate (CAS 10049-21-5, Merck, Darmstadt, Germany) were used.

### 2.2. Organisms and Sample Preparation

*Artemia salina* (aged 4 weeks) were obtained from the Marine Culture Laboratory (registration number ES110280000312) at the Marine and Environmental Sciences Faculty (University of Cádiz). The organisms were treated in accordance with the ethical guidelines of the European Union Council (Council Directive 86/609/EEC) and the Bioethical Committee of the University of Cádiz (Spain). Specimens were anaesthetized by immersion in ice.

The tests yielded the following results: pH = 7.49–7.63, dissolved oxygen = 75.4–98.3%, conductivity = 48.20–49.37 mS/cm and temperature = 20.55–21.22 °C. There were no significant variations in the parameters of the aquariums. Acclimation to laboratory conditions prior to the tests: After 28 days of decapsulation of the *A. salina*, the organism was kept for 48 h under the same conditions as during the laboratory tests but fed *ad libitum.*

### 2.3. Exposure Experiments

#### 2.3.1. MP Intake Bioassay

*A. salina* specimens were exposed to nominal concentrations of 0.26, 0.69 and 1.6 mg/dm^3^ of polyvinyl chloride in seawater, in 25 mL flasks, with each containing 10 adults. These concentrations were selected based on the results obtained by other authors [45,46]. Three replicates of each concentration, as well as a control concentration, were used. The tests were conducted according to the guidelines of the Organisation for Economic Co-operation and Development (OECD) chemical test guideline 202 [47], testing the dead/immobilization of *A. salina*. To reach the stable microplastic concentrations, a stock suspension of microplastics was added, previously stirred. MP concentrations were not quantified. After the 48-h test period, a visual analysis of the digestive system of the organisms exposed to the MP was observed under an optical microscope to check whether the specimens had ingested MP.

#### 2.3.2. Pesticides, Pharmaceutical Products, MPs and Reference Toxicity Test

Potassium dichromate (K_2_Cr_2_O_7_) was used as a reference test. The concentrations used were 80, 40, 20, 10, 5, 2.5 and 1.25 mg/dm^3^, as well as a seawater control. Glass flasks of 25 cm^3^ were used. There were 3 replicates for each treatment and 10 organisms in each flask. The exposure duration was 48 h at 21 °C and 16 h of light/8 h of darkness. *A. salina* were not fed. At the end of the test, the surviving individuals were counted; if they did not show any movement, they were considered dead. The concentrations of simvastatin and carbamazepine were 12.03, 10.03, 8.35, 6.96, 5.80 and 52.08 mg/dm^3^; and 43.40, 36.17, 30.14 and 25.16 mg/dm^3^ in seawater, respectively. Following these tests, the concentrations of triclosan and chlorpyrifos were 12, 6, 3, 1.5, 0.75 and 0.0312 mg/dm^3^; and 0.0156, 0.0078, 0.0039 and 0.00195 mg/dm^3^, respectively.

The nominal MP concentration 0.26 mg/dm^3^ was chosen based on a bibliographic review [45] and was considered environmentally relevant. Acetone was used as the solvent due to the hydrophobic nature of chemical compounds. A control with seawater and another with acetone were used in all the tests. Eight semi-static toxicity tests with three replicates were undertaken: simvastatin, simvastatin–MP, carbamazepine, carbamazepine–MP, TCS, TCS–MP, CPF and CPF–MP. Ten specimens of *Artemia salina* (aged 4 weeks) of approximately 8 mm of size were included in each flask. The solvent concentration in the acetone control and chemical component-only exposures was <0.001% *v/v*. In the MP tests, the polyvinyl chloride stock suspension (0.26 mg/dm^3^) was added. 

Surviving and dead/immobilized organisms were counted. Surviving *A. salina* specimens were preserved frozen at –80 °C, until the time of analysis. In addition, before conservation, living organisms from the tests with MPs were observed under an optical microscope to check whether they had ingested the MPs.

#### 2.3.3. Sample Preparations

After euthanasia by ice, the samples were stored at –80 °C until processing. The surviving *A. salina* from each concentration were rapidly thawed and homogenized cold at the rate of 100 μL of phosphate buffer (0.1 M pH 7.4), using an Ultraturrax homogenizer and centrifuged at 9000 g for 30 min at 4 °C. The supernatants of each sample were then collected.

### 2.4. Enzymatic Assays

ChE activity was measured according to the Ellman et al. (1961) [48] method, adapted for use with a microplate reader as previously described by Albendín et al. (2017) [49]. Briefly, 50 µL of sample was mixed with a mixture containing 30 cm^3^ of 0.1 M phosphate buffer (pH 7.4), 1 cm^3^ of DTNB 0.01 M (0.27 mM: final well concentration) and 0.2 cm^3^ of ASCh 0.2 M (1.07 mM: final well concentration). The change in optical density over time was recorded at 415 nm for 3 min.

The protein content of the samples was measured using Bradford’s (1976) [50] method, adapted to microplates using bovine serum albumin as the standard. All enzyme and protein determinations were prepared in triplicate at 25 °C. ChE activities were expressed as nmol hydrolyzed substrate/min/mg of protein. The specific activities are reported below as the mean ± SD.

### 2.5. Data and Statistical Analysis

The mortality data were treated with the program of the Environmental Agency of the United States (EPA-USA), probit method. The values of CL_50_ for all the chemical compounds individually and associated with the MPs were studied.

Cholinesterase activity data were statistically analysed using the IBM SPSS Statistics (IBM, Madrid, Spain) for Windows Version 23 program. Assumptions regarding the data’s normality and homogeneity of variance were tested by using the Shapiro–Wilk test and Levene’s test, respectively. When the assumption of normality was not satisfied, the Kruskal–Wallis test was used to determine whether there was any significant difference and the Tukey or the Mann–Whitney U test was used to determine between which groups there was a significant difference. Statistical differences between groups were accepted for *p*-values lower than 0.05.

When the assumption of normality was followed, a one-way analysis of variance (ANOVA) was employed to assess the differences of AChE activity among the different chemical compounds, followed by Dunnet’s post hoc comparison test.

## 3. Results

### 3.1. Toxicity Reference Test

The LC_50_ value of K_2_Cr_2_O_7_ during the 24-h duration of the test was 11.80 mg/dm^3^. The values obtained for the lower and upper confidence limits, both of 95%, were 8.75 and 16.07 mg/m^3^, respectively.

The database of the State Agency for the Protection of the Environment (EPA) has established that the LC_50_ value for this toxic substance in acute toxicity tests on invertebrates should be in the range of 0.067–59.90 mg/dm^3^. When the value obtained for K_2_Cr_2_O_7_ was found, i.e., LC_50_ of 11.80 mg/dm^3^, which is within this range, it was determined that the quality of the *A. salina* population was optimal for carrying out subsequent tests with the substances.

### 3.2. Toxicity Test: Pharmaceuticals, Pesticides and MPs

**Figure 1:**
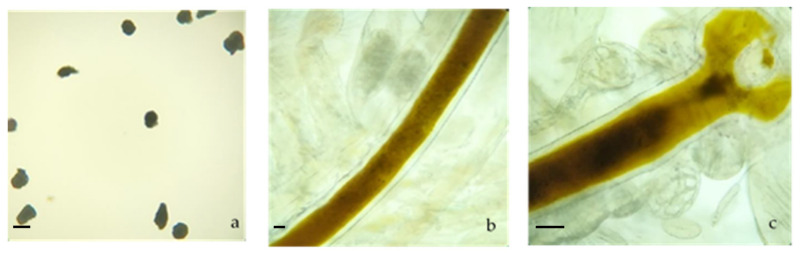
MPs and *Artemia salina*: (**a**) MPs used in the tests, (**b**) *Artemia salina* control without MPs after 48 h and (**c**) *Artemia salina* mouth control without MPs after 48 h. Scale bar (**a**–**c**): 200 µm.

**Figure 2:**
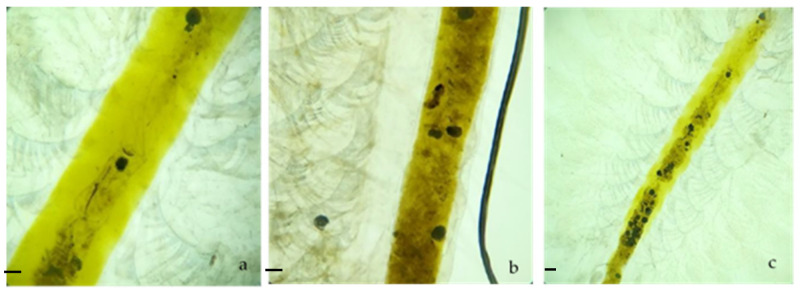
*Artemia salina* after 48 h of exposure to different MP concentrations: (**a**) 26 mg/dm^3^ of MPs, (**b**) 0.69 mg/dm^3^ of MPs and (**c**) 1.5 mg/dm^3^ of MPs. Scale bar (**a**–**c**): 200 µm.

In Figure 1, the samples of MPs and digestive tract of *A. salina* in the control group are observed. *Artemia salina* ingested MPs during the 48 h exposure experiments at all the concentrations exposed (0.26, 0.69 and 1.5 mg/dm^3^; Figure 2), as particles were detected in the digestive tract. These nominal concentrations were used to check whether the MPs indeed accumulated in *A. salina*. In the subsequent tests, the lowest concentration was used, so it can be considered ecologically relevant [51].

The organisms that had ingested MPs were counted. No mortality was recorded in any of the concentrations or in the seawater control during the test. The number of *A. salina* with MPs increased with concentration (Table 1). In the lowest concentration used, 16.6% of individuals had ingested MPs.

MPs were found in the digestive tract of the surviving *A. salina* of the simvastatin and MP test; these were detected in 58 of the 127 specimens exposed to MPs that survived. In the carbamazepine and MP test, these were found in 71 of the 130 that survived. Meanwhile, in the CPF + MP and TCS + MP test, these were not observed.

**Figure 3:**
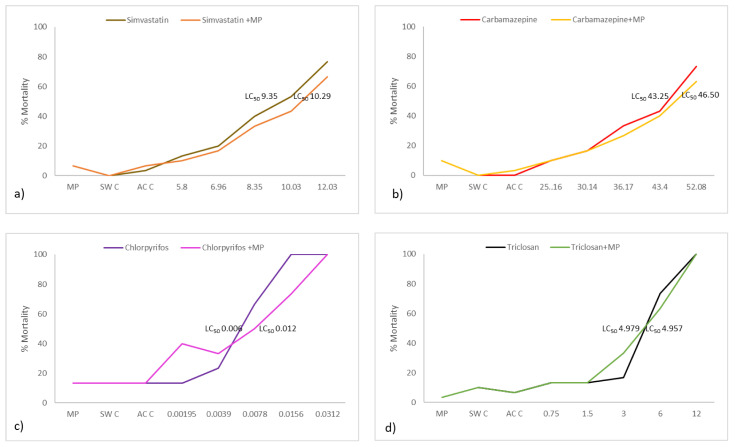
Mortality rate of *A. salina* after 48 h of exposure to different contaminant concentrations (mg/dm^3^): (**a**) simvastatin and simvastatin + MP; (**b**) carbamazepine and carbamazepine + MP; (**c**) CPF and CPF + MP; and (**d**) TCS and TCS + MP. Mean values are represented. SW C: Seawater Control; AC C: Acetone Control.

The mortalities recorded in tests where organisms were exposed to different increasing concentrations of simvastatin, simvastatin–MP, carbamazepine, carbamazepine–MP, TCS, TCS–MP, CPF and CPF–MP, as shown in Figure 3.

The toxicity acute test showed lower mortality in organisms exposed to simvastatin and MP together compared to simvastatin alone. At the lowest concentrations (5.80 and 6.96 mg/dm^3^), the mortality difference was slightly smaller in this test, becoming more marked in the concentration of 8.35 mg/dm^3^. At the highest concentrations (10.03 and 12.03 mg/dm^3^), the difference in mortality between the two assays was greatest, being 10% in both concentrations. In the visual controls that were carried out during both tests, no alteration was observed, neither in the physiology nor in the behavior of the organisms. The same mortality rates (10% and 16.66%, respectively) occurred in *A. salina* exposed to concentrations of 25.16 and 30.14 mg/dm^3^ in the tests with carbamazepine only and carbamazepine associated with MPs. The greatest mortality difference (10%) between the treatments occurred in the concentration of 52.08 mg/dm^3^ of carbamazepine.

The organisms did not manifest changes in physiology, even though they did in their behavior. *A. salina* survivors at concentrations of 30.14 and 36.17 mg/dm^3^ in both assays exhibited slow mobility with respect to the control, including alternating periods of immobility with periods of fast swimming movements with respect to the control. At the highest concentrations of 43.40 and 52.08 mg/dm^3^ (carbamazepine and carbamazepine + MP), *A. salina* did not show mobility and only reacted when mechanically stimulated with a glass rod.

Comparing the CPF treatments with and without MP, at the lowest concentrations (0.00195 and 0.0039 mg/dm^3^), we see that the percentage of mortality was lower in the chlorpyrifos test versus chlorpyrifos and associated MP, but at higher concentrations (0.0078 and 0.0156 mg/dm^3^), the pattern changed, mortality being lower for chlorpyrifos and MP. At the concentration of 0.0312 mg/dm^3^, there was 100% mortality in both tests.

The mortality at 0.75 and 1.5 mg/dm^3^ did not differ in the triclosan test with and without MP. At a concentration of 12 mg/dm^3^, there was 100% mortality in both tests. In general, at the highest concentrations, mortality was slightly lower in tests where the toxicant was associated with MP, but they were not statistically different.

According to the OECD guide [47], the mortality of controls cannot exceed 10% in order for the test to be considered valid. Given that none of the four tests performed in this study exceeded this percentage, they could be regarded as such.

The LC_50_ values and 95% confidence intervals for *A. salina* exposed to the different substances are presented in Table 2.

### 3.3. Enzymatic Assay

**Figure 4:**
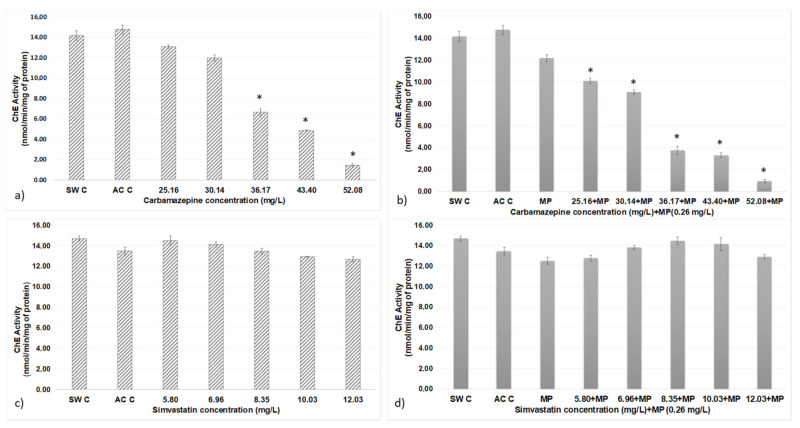
ChE activity of *A. salina* after 48 h of exposure to different contaminant concentrations: (**a**) simvastatin, (**b**) simvastatin + MP, (**c**) carbamazepine and (**d**) carbamazepine + MP. Error bars represent standard deviations. * Significantly different between controls and other treatments (*p* > 0.05).

The results regarding cholinesterase activity in simvastatin, carbamazepine and both in association with MP are shown in Figure 4.

In the test with carbamazepine, statistically significant differences were obtained between the seawater and acetone controls at concentrations of 36.17, 43.30 and 52.08 mg/dm^3^; between the concentration of 25.16 mg/dm^3^ and the concentrations of 36.17, 43.30 and 52.08 mg/dm^3^; between the concentration of 30.14 mg/dm^3^ and the concentrations of 36.17, 43.30 and 52.08 mg/dm^3^; between the concentration of 36.17 mg/dm^3^ and that of 52.08 mg/dm^3^; and between the concentration of 43.30 mg/dm^3^ and that of 52.08 mg/dm^3^. 

In the tests with carbamazepine + MP, an increase in the inhibition of cholinesterase activity was observed versus only with carbamazepine treatments. After statistical analysis of the data, statistically significant differences were obtained between the controls for seawater, acetone and all concentrations of MP-associated carbamazepine that were used in the test. MPs showed significant differences with concentrations of 25.16, 30.14, 36.17, 43.40 and 52.08 mg/dm^3^. These differences were also determined between the concentration of 36.17 mg/dm^3^ and those of 25.16 and 30.14 mg/dm^3^, between the concentration of 43.30 mg/dm^3^ and those 25.16 and 30.14 mg/dm^3^ and between the concentration of 52.08 mg/dm^3^ and 25.16 and 30.14 mg/dm^3^. 

In addition, the results obtained between the same concentrations of both assays were compared (simvastatin and MP associated with simvastatin), revealing no statistically significant differences. 

**Figure 5:**
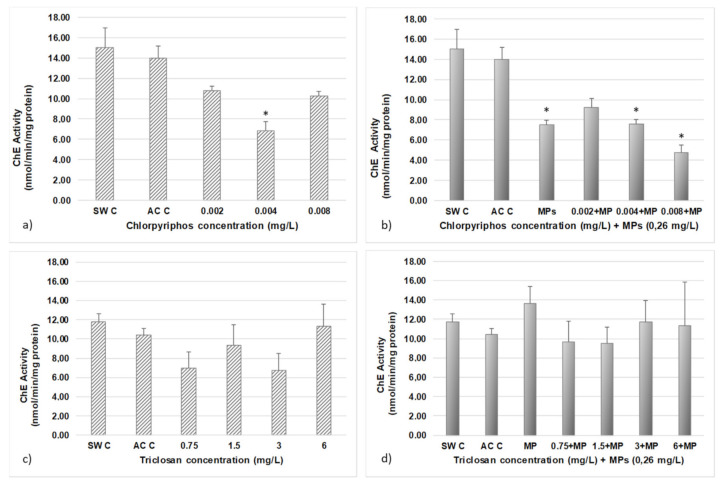
ChE activity of *A. salina* after 48 h of exposure to different contaminant concentrations: (**a**) CPF, (**b**) CPF + MP, (**c**) TCS and (**d**) TCS + MP. Error bars represent standard deviations. * Significantly different between controls and other treatments (*p* > 0.05).

The results of cholinesterase activity in CPF, TCS and either in association with MP are shown in Figure 5. 

The concentration value of 0.008 mg/dm^3^ CPF was considered abnormally high (possibly due to biological variability), so this result is disregarded in the Discussion section below.

Statistically significant differences in CPF + MP treatments were observed between the MP and the controls, between the concentrations of 0.004 mg/dm^3^ and the 0.008 mg/dm^3^ + MP, between 0.002 mg/dm^3^ and 0.004 mg/dm^3^ and 0.008 mg/dm^3^ + MP concentration, and between 0.002 mg/dm^3^ + MP and 0.008 mg/dm^3^ + MP.

No differences were observed between equal concentrations of 0.002 and 0.004 mg/dm^3^ CPF with and without MP.

## 4. Discussion

Pesticides, pharmaceuticals and MPs are environmental contaminants whose hazardous potential (especially in the case of the latter) have been ignored until relatively recently. Nevertheless, they can have direct effects on organisms, as well as act as vectors of other pollutants, potentially modifying their toxicity and bioavailability [32,33]. In this study, we observed the variability of responses between different pollutants with and without MP in the crustacean *A. salina*.

Among the values obtained for LC_50_, simvastatin was found to be most toxic (LC_50_ of 9.348 mg/dm^3^), followed by the combination of simvastatin and MP (LC_50_ of 10.294 mg/dm^3^), carbamazepine (LC_50_ of 43.225 mg/dm^3^) and, finally, simvastatin with MP (LC_50_ of 46.503 mg/dm^3^). The presence of MPs in the treatments containing them showed a slight increase in LC_50_.

The LC_50_ of simvastatin has been determined in different crustaceans. Dahl et al. (2006) [52] observed a 96-h LC_50_ of 810 µg/dm^3^ for *Novanapis spinipes*; however, Key et al. (2008) [53] observed a 96-h LC_50_ of 1.18 mg/dm^3^ for larvae of *Palaemonetes pugio* and more than 10 mg/dm^3^ for adult specimens of the same species, a value that was very similar to that obtained for *A. salina* (9.348 mg/dm^3^). In fish, Ribeiro et al. (2015) [54] studied the effects of simvastatin on zebrafish (*Danio rerio*), establishing an LC_50_ of 5 mg/dm^3^ for zebrafish embryos. Another study carried out by Key et al. (2009) [55] determined an LC_50_ of 2.68 mg/ dm^3^ for *Fundulus heteroclitus*.

In *A. salina*, statistically significant differences were not recorded between the measures of cholinesterase activity in the control groups and the groups exposed to the different concentrations of simvastatin and simvastatin associated with MP tests. Similar results were observed by Key et al. (2009) [55] regarding simvastatin exposure for 96 h in *Fundulus heteroclitus*, although reduced cholinesterase activity declined slightly, if not significantly. In crustaceans, the test carried out by Key et al. (2008) [53] showed an LC_50_ of simvastatin in the range of 0.001 to 10 mg/dm^3^, which did not cause alterations in the acetylcholinesterase activity of larvae and adults of the shrimp *Palaemonetes pugio*. Considering that simvastatin is a commonly used and highly prescribed drug whose discharge into the aquatic environment has increased in recent years, it is reasonable to expect that its presence in the environment tends to increase and consequently enhance the risk of undesirable effects on the most sensitive species. For this reason and given this study’s results, it is advisable to continue investigating the sublethal effects that this drug can cause, carrying out trials with other taxa and with organisms at different stages of development, using different biomarkers to assess the possible effects produced and increasing the time of exposure to the drug through chronic toxicity tests.

In the case of carbamazepine, the 48-h LC_50_ for *Artemia salina* was 43.225 mg/dm^3^. In other crustaceans, such as *Daphnia magna*, Kim et al. (2007) [56] estimated a 96-h LC_50_ of 76.3 mg/dm^3^. A very similar concentration was obtained by Ferrari et al. (2004) [57]: a 48-h LC_50_ of 77.7 mg/dm^3^ in the same organism. These concentrations differ from those obtained in this work; hence, *A. salina* seems to be a more sensitive organism to carbamazepine than *Daphnia magna*. For the association of carbamazepine with MP, an LC_50_ of 46.503 mg/dm^3^ was obtained, which was higher (by 3.303 mg/dm^3^) than carbamazepine alone. A similar pattern was obtained with simvastatin. Besides, we observed that toxicity decreased in the combination of pharmaceuticals and MP. However, Brandts et al. (2018) [58] determined that exposure to carbamazepine associated with nanoplastics (polystyrene) induced a significant negative regulation in genetic expression in *Mytilus galloprovincialis* compared to exposure to carbamazepine only. In addition, studies carried out by Gambardella et al. (2017) [59] showed that exposure to MP (polystyrene) caused neurotoxic effects in *nauplii* of *Artemia franciscana* and *Amphibalanus Amphitrite*. Moreover, the ChE activity in carbamazepine and carbamazepina + MP exposed to *A. salina* was significantly inhibited in our study. Similar results have been obtained by other authors with different species of invertebrate organisms. For instance, Siebel et al. (2010) [60] observed via an in vitro test that carbamazepine inhibited acetylcholinesterase activity in the brain of zebrafish (*Danio rerio*). In crustaceans, Nkoom et al. (2019) [61] reported a significant decrease in acetylcholinesterase activity in *Daphnia magna* specimens exposed to concentrations of 5 and 100 µg/L, as well as an ability to bioconcentrate in the aqueous medium under laboratory conditions. The ChE activity recorded in this test (carbamazepine + microplastic) was slightly lower than that obtained in the isolated carbamazepine test, but no statistically significant differences were obtained using the same concentrations in the two tests. 

On the other hand, the smaller LC_50_ values from lowest to highest were: CPF, CPF + MP and finally TCS + MP and TCS, which were similar. The value of 48-h LC_50_ for TCS (4.979 mg/dm^3^) obtained in this study was higher than associated values in the literature, probably related to the age of the crustaceans. For instance, Xu et al. (2015) [62] reported a 24-h LC_50_ of 0.171 mg/dm^3^ in a TCS toxicity test on *A. salina* (*nauplium*). Iannacone et al. (2016) [63] studied antimicrobial toxicity in *Artemia franciscana* (*nauplii* phase II), yielding a 48-h LC_50_ of 0.72 mg/dm^3^. Perron et al. (2012) [23] investigated the effect of TCS on the invertebrates *Ampelisca abdita* and *Americamysis bahia*, reporting 48-h LC_50_ values of 0.0913 and 0.0956 mg/dm^3^, respectively. Regarding the effect of TCS in zebrafish (*Danio rerio*), Oliveira et al. (2009) [64] reported 96-h LC_50_ values of 0.42 mg/dm^3^ for embryos/larvae and 0.34 mg/dm^3^ for adults. These authors also examined variations in the values of biomarkers, including cholinesterase activity. They did not observe decreased enzymatic levels, similar to our study, where significant differences were not seen between the various concentrations tested and the control.

Sybeg et al. (2017) [65] tested with *Acartia tonsa* (adult) exposure to TCS alone and polyethylene (500 MP/cm^3^), yielding a 48-h LC_50_ of 0.1579 mg/dm^3^ for TCS and 0.1096 mg/dm^3^ for TCS + MP. With these data, the authors concluded that the mixture of TCS + MP was more toxic than TCS alone. These data disagree with our results, where no difference was observed between the LC_50_ of the TCS and TCS + MP.

Regarding *A. salina*, the 48-h LC_50_ values of 0.006 mg/dm^3^ and 0.012 mg/dm^3^ for CPF and CPF + MP, respectively, are in the range of those found by other authors in adults and juveniles. For instance, Osuna et al. (1997) [66] evaluated the toxicity to CPF in *Penaeus* sp. (juveniles), observing a 48-h LC_50_ of 0.00207 mg/dm^3^. Galindo et al. (1996) previously indicated a 48-h LC_50_ of 0.0048 mg/dm^3^ in a test with the crustacean *Penaeus vannamei* (juveniles) exposed to CPF.

Varó et al. (1998) [67] exposed to CPF on *nauplii* of different species of the genus *Artemia* sp. and observed differences in sensitivity to the toxic substance, indicating that the most sensitive species was *Artemia salina* (24-h LC_50_ from 0.95 to 5.12 mg/dm^3^). They also described differences that were not only based on the species tested (*A. salina, A. persimilis, A. franciscana* and *A. parthenogenetica*), but also between strains of the same species. Accordingly, these authors exposed to CPF in *Artemia parthenogenetica*, adult fish *Gambusia affinis* and *Aphanius iberus* and observed increased resistance to CPF in the genus *Artemia* compared to the two fish species (48-h LC_50_ for *G. affinis* of 0.5 mg/dm^3^ and 48-h LC_50_ *for A. iberus* of 0.0386 mg/dm^3^). In addition, the genus *Artemia* showed different sensitivity to the toxic substance according to the degree of development or intraspecific variability (*nauplii* 24-h LC_50_: juveniles, 3.9 ± 0.9 mg/dm^3^; adults, 0.08 ± 0.01 mg/dm^3^).

Baek et al. (2015) [68] evaluated the effect of CPF, among other biocides, on *A. salina* (*nauplium*), yielding a 24-h LC_50_ of 2.032 (0.416–6.891 mg/dm^3^), within the range of Varó et al.’s (1998) [67] findings. In the same study, acetylcholinesterase activity was studied, comparing newborn *nauplii* with 48-h *nauplii*. The former was found to exhibit a greater decrease in cholinesterase activity than the 48-h *nauplii*, following exposure to CPF for 24 h, rendering this a very useful marker for predicting signs of contamination by this toxic substance in the environment. The data from these studies indicate that there are differences in sensitivity, not only among species, but also depending on the stage of development. Adults and juveniles seem to be more sensitive than *nauplii*, so the choice of biomarker and developmental stage is of great importance in experiments to ensure a higher safety range. In our study, the LC_50_ data regarding *A. salina* were more in line with the values provided by these authors working with adults and juveniles. 

The association of CPF with MP was not reflected in an increase in toxicity with CPF, as the LC_50_ value was higher. This toxicity-reducing effect has been mentioned by other authors [18], who have indicated that CPF adsorption to MP can reduce toxicity’s effect on algal growth (*Isochrysis galbana*). 

On the other hand, we observed a significant decrease in cholinesterase activity by the CPF + MP test, just as Oliveira et al. (2013) [69] and Luís et al. (2015) [45] found that acetylcholinesterase activity in samples of *Pomatoschistus microps* exposed to MP (polyethylene) to a 96-h test was lower than in a control. However, in the TCS + MP test, no significant differences were found, as has also been described by other researchers working with aquatic organisms [64,70]. 

Organophosphate substances (CPFs) are known as prototype inhibitors of this enzyme. In fact, the specific activity of cholinesterase in *A. salina* decreases as concentrations of CPF increase [67]. Nevertheless, in this work, no statistically significant differences in cholinesterase activity were observed between equal concentrations of CPF with and without MP.

The results obtained indicate that the presence of MP in the medium reduces the toxicity, considering the LC_50_ values that may be related to the low MP intake detected in our preliminary test, since the concentration used (0.26 mg/dm^3^) was lower. This concentration was related to the concentration of MPs observed in the environmental. However, inhibition of cholinesterase activity of *A. salina* exposed with CPF, simvastatin and CPF + MP, simvastatin + MP in a concentration-dependent ratio showed a synergistic effect of MP+ other contaminants, probably due to their possible role as vehicles of exposure. Eder et al. (2021) [71] reviewed studies showing that the toxicity of pharmaceuticals and other particles and chemicals increased when exposure was combined with MP. Among the adverse effects, they distinguished the same biomarker studied in our work, the inhibition of cholinesterase activity. The synergistic effect could be determined by the sorption behavior of organic contaminants to MP that, according to Wang et al. (2020) [72], are partitioning, surface sorption and pore filling, as well as by the properties of the MP, organic contaminants and aquatic conditions. Thus, the interaction of MPs with contaminants can result in aggregation, reduced bioavailability and alteration in the toxicity to the organism [73].

## 5. Conclusions

In this study, the adverse effects of pharmaceutical compounds with associated MPs and pesticides with associated MPs were studied. Regarding 48-h LC_50_ in *A. salina*, the MPs did not potentiate the adverse effects of pollutants when they were co-exposed; however, cholinesterase activity decreased more if the pollutant (drug or pesticide) was in contact with the MP than without them, indicating a cellular effect.

The concentration of MP exposed to *A. salina* was used by other authors for ecologically relevant studies, but the preliminary studies in this work with MP ingestion by organisms showed that, if the concentration of MP increased, the number of *A. salina* that incorporated MPs also increased, and this could suggest, that if the MP concentration increases in the environment, toxicity in the organism can also increase.

## Figures and Tables

**Table 1:** Quantification of MPs in the digestive tract of *Artemia salina.* Number of organisms in which MP was detected (MP)/number of organisms in which MP was not detected (No MP).

	Sample Replication			Total
MP	MP/No MP	MP/No MP	MPs/No MP	MPs/No MP
0.26 mg/dm^3^	2/8	2/8	1/9	5/30
0.69 mg/dm^3^	4/6	5/5	2/8	11/30
1.5 mg/dm^3^	3/7	7/3	9/1	19/30

**Table 2:** Forty-eight-hour LC_50_ values and confidence intervals for adult *A. salina* exposed to different toxicants (simvastatin, simvastatin + MP, carbamazepine, carbamazepine + MP, CPF, CPF + MP, TCS and TCS + MP).

Emergent Contaminants	LC_50_ (mg/dm^3^)	95% Limits
Simvastatin	9.35	8.58–10.43
Simvastatin + MP	10.29	9.34–12.02
Carbamazepine	43.25	39.59–49.04
Carbamazepine + MP	46.50	41.95–55.72
CPF	0.006	0.002–0.009
CPF + MP	0.012	0.001–0.018
TCS	4.979	2.096–6.0872
TCS + MP	4.957	2.431–7.093
